# Birth Weight and Nutrient Restriction Affect Jejunal Enzyme Activity and Gene Markers for Nutrient Transport and Intestinal Function in Piglets

**DOI:** 10.3390/ani11092672

**Published:** 2021-09-11

**Authors:** Michael O. Wellington, Lucas A. Rodrigues, Qiao Li, Bingqi Dong, Josiane C. Panisson, Chengbo Yang, Daniel A. Columbus

**Affiliations:** 1 Prairie Swine Centre, Inc., Saskatoon, SK S7H 5N9, Canada; michael.wellington@usask.ca (M.O.W.); lucas.rodrigues@usask.ca (L.A.R.); josiane.panisson@usask.ca (J.C.P.); 2 Department of Animal and Poultry Science, University of Saskatchewan, Saskatoon, SK S7N 5A8, Canada; 3 Department of Animal Science, University of Manitoba, Winnipeg, MB RT3 2N2, Canada; liqiao2017hzau@163.com (Q.L.); bingqi.dong@umanitoba.ca (B.D.); chengbo.yang@umanitoba.ca (C.Y.)

**Keywords:** low birth weight, normal birth weight, neonatal piglets, intestinal development, gene expression, normal nutrition, restricted nutrition

## Abstract

**Simple Summary:**

Birth weight and nutrient utilization are thought to have significant effects on intestinal development in neonatal pigs. The present study evaluated the impact of low and normal birth weight with and without nutrient restriction during the neonatal period on jejunal development. The results observed suggest that during the first 28 d of life, birth weight had greater effects on intestinal development than nutrient level, however, at d 56, the nutrient level was a significant contributor to intestinal function and enzyme activity compared to birth weight. Taken together, both birth weight and nutrient restriction have effects on intestinal development, but may have a greater impact in early life (d 28).

**Abstract:**

Significant variation in the birth weight of piglets has arisen due to increased sow prolificacy. Intestinal development and function may be affected by birth weight. Low birth weight (LBW) pigs may also have reduced feed intake, leading to further impairment of intestinal development. The objective of this study was to examine the intestinal development pattern of LBW and normal birth weight (NBW) piglets with normal nutrition (NN) or restricted nutrition (RN) in the pre-weaning period. Jejunal intestinal samples were analyzed for target gene expression and enzyme activity at d 28 (weaning) and d 56 (post-weaning). At d 28, excitatory amino acid transporter (EAAC1) and sodium-dependent neutral amino acid transporter (B^0^AT1) were downregulated in LBW compared to NBW pigs (*p* < 0.05). On d 56, B^0^AT1 and ASCT2 (glutamine transporter) were downregulated in RN compared to NN pigs (*p* < 0.05), regardless of birth weight. Peptide transporter 1 (PepT1) expression was downregulated in LBW compared to NBW pigs at 28 d (*p* < 0.05), with no effects of treatments at 56 d. Sodium-glucose transporter-1 (SGLT1) was upregulated in NBW-NN compared to LBW-NN pigs (*p* < 0.05) at 28 d. Alkaline phosphatase (ALP) was upregulated in LBW-RN at d 28. At d 56, claudin-3 (CLDN-3) and Zonular occludin-1 (ZO-1) were upregulated in NN compared to RN pigs (*p* < 0.05). There were no treatment effects on ALP, maltase, or sucrase activity at 28 d. However, at 56 d, ALP was upregulated in NBW-NN pigs while sucrase activity was upregulated in NN pigs (*p* < 0.05). The results demonstrate differences in jejunal gene expression associated with birth weight, with reduced gene expression of amino acid transporters (PepT1, EAAC1, B^0^AT1) in LBW compared to NBW pigs (*p* < 0.05). While neonatal nutrient restriction had minimal effects at 28 d and d 56 for tight junction protein transcript abundance, neutral amino acid transporter abundance was upregulated in NN pigs compared to RN piglets (*p* < 0.05).

## 1. Introduction

There is significant variation in the birth weight of piglets within the same litter, which is primarily related to an increase in sow prolificacy (i.e., increased litter size) [1,2]. The survival and development of neonatal piglets are associated with birth weight [3,4], with piglets born at low birth weight (LBW) at higher risk of poor growth and development, including inadequate intestinal development and function. For example, Tao et al. [5] investigated the effects of birth weight on the gastrointestinal barrier function of pigs at 90 d of age and reported increased intestinal damage, decreased antioxidant capacity, increased abundance of proinflammatory cytokines, and inhibition of tight junction proteins expression in LBW compared to normal birth weight (NBW) pigs. Moreover, it has been shown that the maturation of digestive function was delayed in LBW pigs, regardless of the stress associated with weaning transition [6]. In addition to their small size, LBW piglets may have reduced access to nutrition in the pre-weaning period due to competition with larger littermates [7,8], potentially exacerbating the negative effects of LBW on intestinal function, as sufficient nutrient intake is critical for intestinal development [9]. Inadequate intestinal development will lead to short- and long-term complications, including poor nutrient digestion and absorption, reduced intestinal barrier function, and increased susceptibility to pathogens [10,11], as well as reduced overall animal performance (i.e., growth). While the development of neonatal pigs has been widely studied, the independent and interactive effects of birth weight and nutrient restriction on intestinal development are not well known. 

We previously developed and validated a model of birth weight and neonatal undernutrition, which demonstrated a modified intermittent suckling protocol as an effective strategy to induce nutrient restriction in pigs [12]. Since our model identified major differences in performance and organ development at 56 d, as influenced by birth weight category (BWC) but not neonatal nutrient restriction, it is important to understand the specific effects of BWC and nutrient restriction on intestinal development and function. 

Therefore, the objective of this study was to examine jejunal enzyme activity and gene expression of nutrient transporters and indicators of intestinal barrier function in LBW and NBW piglets with either normal pre-weaning nutrition (**NN**) or restricted nutrition (**RN**) on jejunal gene expression and enzyme activity. We hypothesized that both LBW and NR would negatively affect intestinal development and function.

## 2. Materials and Methods

The protocols used in this study were approved by the Animal Research Ethics Board of the University of Saskatchewan (Animal Use Protocol #20190042) and followed the Canadian Council of Animal Care guidelines for the care and use of farm animals in research.

### 2.1. Animals, Housing, and Experimental Design

Details of the experimental procedure have been outlined previously [12]. Briefly, a total of 14 sows and 112 piglets (Camborough Plus × C3378; PIC Canada Ltd., Winnipeg MB, Canada) were housed in farrowing crates at the Prairie Swine Centre (Saskatoon, SK, Canada) over 4 blocks (farrowing group) and fed a common commercial lactation diet (Masterfeeds, Saskatoon, SK, Canada). After farrowing, all piglets in the litter were individually weighed and identified as LBW or NBW based on previously established weight ranges in this population of pigs [13], with piglets of <1.5 kg initial body weight considered LBW. Postnatal nutrient restriction (RN) was induced in 4 target piglets per litter (2 LBW and 2 NBW, balanced for sex) through isolation from the sow for 6 h/d from 0800–1400 h from d 3 post-farrowing to weaning (d 28), based on a modification of previously described methods [14,15]. All other piglets were allowed unrestricted access to the sow [normal nutrition (NN)]. No additional feed was provided to the piglets before weaning. At the end of the 28-d suckling period, piglets were weaned and housed in groups of 3–6/pen and fed a common commercial nursery diet (Masterfeeds) from d 29–56 post-weaning. At d 28 and 56 of the study, 8 piglets/treatment (2 piglets/treatment per block) were randomly selected (balanced for sex) and euthanized with an overdose of isoflurane (oxygen flow at 1 L/min with 5% isoflurane) followed by exsanguination. After evisceration, sections of the small intestine (jejunum) were sampled and immediately snap-frozen in liquid nitrogen and then stored at −80 °C until analysis. In all, a total of 16 piglets were euthanized for each treatment (8 piglets/treatment on d 28 and 8 piglets/treatment on d 56) for a total of 64 piglets.

### 2.2. Enzyme Activity Assay

The enzyme activities of intestinal digestive enzymes including intestinal alkaline phosphatase (ALP), maltase, and sucrase were determined. Briefly, approximately 500 mg of pulverized and frozen jejunal tissue samples were thawed in an ice-cold homogenizing buffer consisting of 50 mM D-mannitol and 0.1 mM phenylmethylsulphonyl fluoride (PMSF) at pH 7.4 (Sigma-Aldrich Chemical Co., St. Louis, MO, USA) and homogenized on ice using a polytron homogenizer (Fisher Scientific, Ottawa, ON, Canada). After centrifugation (4000 rpm, 10 min, 4 °C), the protein concentrations of the homogenate suspensions were determined using a Bicinchoninic acid Protein Assay kit (Thermo Fisher Scientific, Waltham, MA, USA). Alkaline phosphatase (EC. 3.4.11.2) activity was assayed according to the method of Engström [16]. Potassium fluoride (Sigma-Aldrich Chemical Co.) was added to inhibit the interference of intracellular acid phosphatase in the intestinal mucosa [17]. Incubations were conducted in a final volume of 0.50 mL containing homogenized tissue suspensions (0.100 to 0.200 mg protein), 2.0 mM potassium fluoride, 5.0 mM MgCl_2_, and 40.0 mM p-nitrophenyl phosphate (Sigma-Aldrich Chemical Co.) at pH 10.5 for 15 min at 37 °C. The enzyme reaction was stopped by adding 0.50 mL of 0.50 M NaOH (Sigma-Aldrich Chemical Co.). The end-product of the enzyme reaction, p-nitrophenol, was measured using a Synergy™ H4 Hybrid Multi-Mode Microplate Reader (BioTek, Winooski, VT, USA) at 400 nm wavelength. Maltase (E.C. 3.2.1.20) and sucrase (E.C. 3.2.1.48) activities were assayed according to a previously established method [18]. Incubations were conducted in a final volume of 0.500 mL containing homogenized cell suspensions (0.200 to 0.300 mg protein), 100 mM maleate buffer (maleic acid dissolved in NaOH), and 60 mM maltose, 60 mM sucrose, or 100 mM amylose, at pH 6.0 and 37 °C for 20 min. Incubations were terminated by adding 70 mM ZnSO_4_. The end-product of both enzyme reactions, D-glucose (Pointe Scientific, Inc., Canton, MI, USA), was measured by spectrophotometric analysis at 500 nm. All enzyme assays were corrected for nonspecific blank readings.

### 2.3. Real-Time Polymerase Chain Reaction (q-PCR)

Tissue samples stored at −80 °C were ground in liquid nitrogen with mortar and pestle and total RNA of the jejunum tissue was extracted by the Trizol reagent procedure (Invitrogen, Carlsbad, CA, USA) according to the manufacturer’s instructions. Total RNA was dissolved in 20 µL RNase-free water and concentration was determined using a NanoDrop 2000 spectrophotometer (Nano-Drop Technologies, Wilmington, DE, USA) with purity ascertained (A260/A280) between 1.8 and 2.0. The total RNA (about 1 µg) from each sample was reversed transcribed into cDNA using iScript cDNA Synthesis Kit (Bio-Rad, Mississauga, ON, Canada) according to the manufacturer’s instructions with a random hexamer primer. Each 20 μL of reaction mix consisted of 0.8 μL of 10 μmol/L primer concentration for each forward and reverse primer, 6.4 μL of nuclease-free water, 10 μL of EVA green supermix (Bio-Rad Laboratories, Hercules, CA, USA) and 2 μL (2 ng/qPCR reaction) of template cDNA. Standard curves were made for each gene using a 5-fold serial dilution of pooled cDNA samples from all experimental treatments. A PCR efficiency between 90% and 110% was accepted as valid for data analysis. The qPCR analysis was performed to quantify the target genes encoding for enzyme (ALP, SI), nutrient transporter (SGLT1, PepT1, EAAC1, ASCT2), and B^0^AT1), and barrier function genes (ZO-1 and Claudin-3), as presented in Table 1. The cyclophilin-A (Cyc-A) gene was used as the housekeeping gene. The relative changes in gene expression levels of genes in the jejunum tissues normalized against Cyc-A were determined by using the 2^−∆∆CT^ method according to Livak and Schmitten [19].

### 2.4. Statistical Analysis

Data were tested for normality and outliers using the PROC UNIVARIATE model and the Shapiro–Wilk test in SAS version 9.4 (SAS Institute Inc., Cary, NC, USA). Outliers were determined as a value ±2 standard deviations away from the treatment mean using the studentized residual analysis. Data were analyzed using the MIXED procedure of SAS with different groups of piglets sampled on d 28 and d 56, analyzed separately as a randomized complete block design with a 2 × 2 factorial arrangement. The fixed effects were (1) birth weight category (BWC) (LBW and NBW), (2) dietary treatment (RN and NN), and (3) their interaction. Block (farrowing group) was included in the model as a random effect. Initially, sex was also included as a fixed effect but was not significant and, therefore, removed from the model. Differences between treatment means were separated by the PDIFF option adjusted for the Tukey test. Significance differences were determined at *p* ≤ 0.05 and a trend toward significance was considered at *p* < 0.10.

## 3. Results

### 3.1. Enzyme Activity 

**Figure 1:**
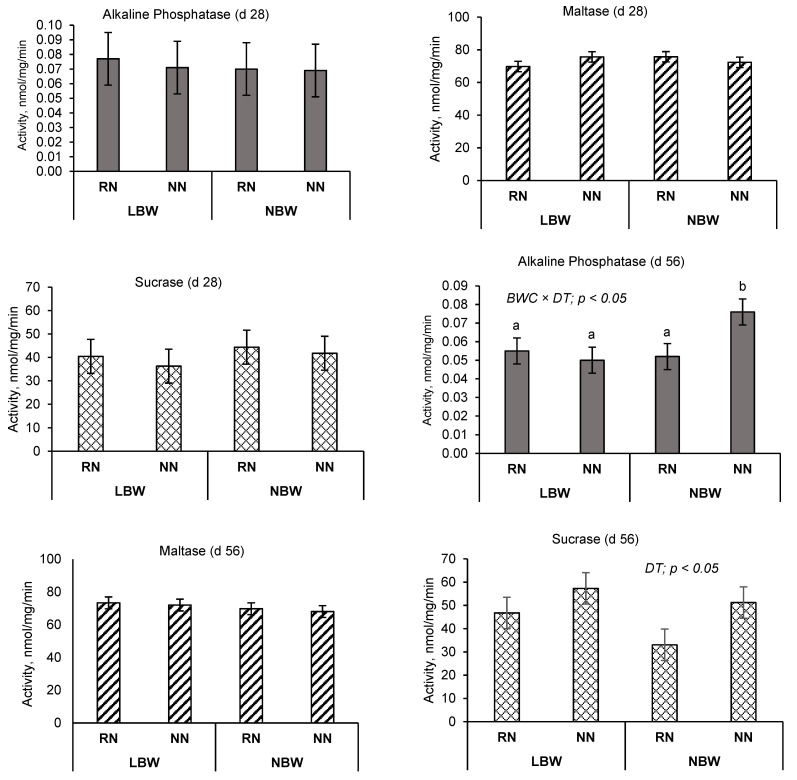
Enzyme activity in jejunal tissue for piglets d 28 and d 56 post-weaning. LBW; Low birth weight, NBW; normal birth weight, RN; restricted nutrition; NN; normal nutrition, BWC; birth weight category, DT; dietary treatment. ^a, b^ Lowercase superscripts are indicative of a significant interaction. There was a significant main effect of dietary treatment (NN vs. RN) on sucrase activity 54.3 vs. 39.9 (*p* < 0.05).

Enzyme activity results are reported in Figure 1, with the foremost panel showing ALP, maltase, and sucrase at d 28 and the other panel showing results for d 56. On d 28, there were no significant effects of treatment on any of the measured parameters. On d 56, there was an interaction between birth weight and nutrient restriction, with the NBW-NN group having greater ALP activity compared to the other treatments (*p* < 0.05). Further, we observed a nutrition level effect on sucrase activity where NN piglets had a significantly higher sucrase activity compared to RN (54.3 vs. 39.9; *p* < 0.05) regardless of BWC. There were no other treatment effects on any other measured enzyme activity.

### 3.2. RT-qPCR of Target Genes 

Table 2 and Table 3 show target gene expression of jejunal tissue samples of piglets at 28 and 56 d, respectively. At weaning (d28), piglets within the NBW category had a significantly higher expression of EAAC1 (3.54 vs. 1.48), B^0^AT1 (2.28 vs. 1.12), and PepT1 (2.56 vs. 1.27) compared to piglets with LBW (*p* < 0.05), with no effect of nutrient restriction at either time point. Further, ZO1, Claudin-3, SI, and ASCT2 were not affected by birth weight or nutrition level. The expression of SGLT1 and ALP was significantly affected by the interaction of birth weight and nutrition level. Specifically, ALP expression was higher in LBW-RN pigs compared to LBW-NN and NBW-RN pigs (*p* < 0.05) but equal to NBW-NN pigs. Expression of SGLT1 was reduced in the LBW-NN compared to NBW-NN pigs (*p* < 0.05) and equal to other treatment groups. At d 56, there were no significant treatment effects on SGLT1, PepT1 or EAAC1. Generally, at d 56, there was a significant effect of the nutrient restriction group (NN vs. RN) on Claudin-3, ZO1, ASCT1, and B^0^AT1. Specifically, NN piglets, regardless of BWC, had greater expression of Claudin-3 (1.84 vs. 0.60), ZO1 (2.70 vs. 0.51), ASCT1 (2.27 vs. 0.34), and B^0^AT1 (3.62 vs. 0.97) compared to RN piglets (*p* < 0.01). There was a significant interaction between BWC and nutrition on ALP and SI expression. Specifically, there was increased expression of ALP and SI in NBW-NN piglets compared to all other treatments (*p* < 0.05).

## 4. Discussion

### 4.1. Birth Weight and Nutrition Effect on Enzyme Activity

Enzyme activity in the gut is important in the digestion and absorption of nutrients, therefore, when enzyme activity is downregulated, there is potential for a negative impact on nutrient absorption. Alkaline phosphatase is directly involved in intestinal immune response and, when the expression is reduced, may predispose the gut to changes in the gut microbiome and intestinal inflammation and permeability [20]. Intestinal inflammation has been reported in LBW [5] as well as nutrient-restricted neonatal pigs [21]. In the present study, we observed no effect of treatment on ALP activity at d 28, whereas ALP enzyme activity was upregulated in NBW-NN compared to the other groups at 56 d. This may be explained by intestinal protective roles of ALP recently reviewed by Lallès [22], which may indicate improved gastrointestinal health as a result of higher birth weight and neonatal nutrient intake. Huygelen et al. [23] reported no effect of birth weight (low vs. normal) on ALP expression in the small intestine of pigs at weaning, which confirms the findings in the present work showing that neonatal nutrient allowance may improve intestinal ALP activity, particularly in the long-term (e.g., 56 d post-weaning). Maltase is a brush border enzyme involved in effectively hydrolyzing α-1,4 and α-1,6 linkages for the digestion of several carbohydrates [24]. Sucrase-isomaltase is also a highly prevalent enzyme for digestion of different dietary carbohydrates, which supports intestinal physiology and immune response [25]. In the present study, we observed no effect of the experimental treatments on maltase or sucrase activities at 28 d, which was consistent with the lack of effect of treatments on SI gene expression. Interestingly, at 56 d, SI activity was upregulated in NN pigs compared to RN pigs, regardless of birth weight. Moreover, it is well known that increased access to milk intake early in life augments gastrointestinal development later in life [26], which may contribute to improved digestion and absorption of carbohydrates. Since the adult pattern of distribution of brush-border carbohydrases is achieved around 8 weeks [27], our findings indicate a better adaptation of NBW-NN pigs to the weaning transition from milk- to cereal-based diet and highlight that neonatal nutrient restriction may play an important role in this establishment. 

### 4.2. Birth Weight and Nutrition Effect on Target Gene mRNA Transcript Abundance

In the present study, we observed an upregulation of ALP gene expression in NBW-NN pigs compared to other treatments at both d 28 and d 56. Alkaline phosphate is known as a regulator of inflammation and may play an active role in intestinal development in early life [15]. Furthermore, sucrase isomaltase gene expression was observed to be upregulated in the NBW-NN group compared to the other treatments on 56, but on d 28, there was a tendency for higher expression in the LBW-RN pigs. Indeed, a recent study reported expression of SI at 14 times higher in NBW compared to LBW piglets and an increased expression of SI with age in NBW but not in LBW [28]. At 28 d, Claudin-3 tended to be upregulated in NBW compared to LBW pigs, with no effects of treatments on ZO-1 and ASCT2. Conversely, at 56 d, Claudin-3 and ZO-1 were upregulated in NN compared to RN pigs. Our findings suggest an improved intestinal physical barrier in NBW pigs around weaning. Interestingly, intestinal barrier function (Claudin-3 and ZO-1 expression) was influenced by neonatal nutrient allowance and not body weight at 56 d. Tissue stability and barrier function are influenced by tight junction protein function, with higher expression being positively correlated with enhanced barrier function [29]. A previous report indicated that milk/colostrum consumption may further enhance tight junction proteins expression [30], which is in line with our results, which demonstrated increased expression of tight junction proteins in NN pigs. This may relate to increased gut barrier protection and improve the ability of pigs to cope with intestinal disturbances post-weaning [31]. At 28 d, EAAC1 (glutamate transporter; [32]) and B^0^AT1 (neutral amino acids transporter; [33]) were downregulated in LBW compared to NBW pigs, which is in agreement with previous studies and suggests that intestinal normal absorptive function and mucosal growth may be decreased in LBW pigs due to decreased amino acid availability for protein synthesis [34,35]. Conversely, at 56 d, there was no effect of treatment on EAAC1 expression, however, both B^0^AT1 and ASCT2 (glutamine transporter) were downregulated in RN pigs compared to NN pigs, with no influence of birth weight. These findings confirm the lack of an independent effect of birth weight on small intestine development, as we have previously reported [12], which is likely dependent on nutrient intake. Moreover, neutral amino acids, including proline [36] and glycine [37], are nutritionally essential for milk-fed pigs and neonatal nutrient restriction may have decreased expression of their transporters later in life. Peptide transporter 1 (PepT1, di- and tripeptides transporter; [38]) has been reported that PepT1 is highly expressed in LBW pigs [39]. However, there is great variability in expression across the different segments of the intestine [40,41], which may explain discrepancies among studies. In the present study, expression of PepT1 was downregulated in LBW compared to NBW pigs at 28 d and showed no effects at 56. This observation suggests that birth weight may influence the absorption of nutrients (e.g., amino acids) in neonatal pigs, likely due to reduced capacity of the gut to feed intake and enzyme secretion. Again, the sodium-dependent glucose transporter 1 (SGLT1, sodium-dependent glucose uptake in the small intestine; [42]) was upregulated in NBW-NN compared to LBW-NN pigs at 28 d, with no effects observed at 56 d. The results indicate that, when piglets fed a normal level of nutrients, their birth weight will have an influence on glucose transport capacity; in this case the results show that pigs with NBW have higher glucose transport capacity than LBW pigs. These results are aligned with another study which reported that SGLT1 expression is influenced by feeding but this may be restricted by birth weight [43].

## 5. Conclusions

In summary, our results showed that jejunal gene expression and enzyme activity at 28 d and 56 were associated with differences in both birthweight and nutrition, with reduced expression of amino acid transporters in LBW compared to NBW pigs. Further, normal birth weight and normal nutrition pigs had upregulated expression of target genes and greater enzyme activity compared to the other groups, indicating that intestinal development may be influenced by both birth weight and nutrition.

## Figures and Tables

**Table 1:** The primer sequences of the target genes and the internal reference gene.

Gene Name	Sequence (5′-3′)	Accession Number	Product Size (bp)
	Enzyme genes		
*ALP*	F: CCACTCCGCGCCACC	XM_021097682.1	76
	R: AAGAGCTCGTGGGTGAAGG		
*SI*	F: TGATAGGCCAGTGAGAGTGC	XM_021069748.1	99
	R: AGAGTTGAGTAAGGCTGCCA		
	Nutrient transporter genes		
*SGLT1*	F: GGCTGGACGAAGTATGGTGT	NM_001164021.1	153
	R: ACAACCACCCAAATCAGAGC		
*EAAC1*	F: GTTCCTGATTGCCGGGAAGA	NM_001164649.1	165
	R: ATGGCGAATCGGAAAGGGTT		
*ASCT2*	F: GCCAGCAAGATTGTGGAGAT	XM_003355984.4	206
	R: GAGCTGGATGAGGTTCCAAA		
*B^0^AT1*	F: AAGGCCCAGTACATGCTCAC	XM_0033559855.4	102
	R: CATAAATGCCCCTCCACCGT		
*PepT1*	F: CATCGCCATACCCTTCTG	NM_214347.1	143
	R: TTCCCATCCATCGTGACATT		
	Barrier function genes		
*ZO-1*	F: GATCCTGACCCGGTGTCTGA	XM_021098896.1	200
	R: TTGGTGGGTTTGGTGGGTT		
*CLDN3*	F: CTACGACCGCAAGGACTACG	NM_001160075.1	123
	R: TAGCATCTGGGTGGACTGGT		
	Internal reference gene		
*CycA*	F: GCGTCTCCTTCGAGCTGTT	NM_214353.1	160
	R: CCATTATGGCGTGTGAAGTC		

ALP; alkaline phosphatase, SI; sucrase-isomaltase, SGLT1; sodium/glucose cotransporter 1, EAAC1; excitatory amino acid transporter 1, ASCT2; glutamine transporter, B^0^AT1; sodium-dependent neutral amino acid transporter, PepT1; peptide transporter 1, ZO-1; zonula occludens-1, CLDN3; claudin 3, CycA; Cyclophilin-A.

**Table 2:** Jejunal nutrient transporter and tight junction mRNA transcript abundance at 28 d post-weaning.

	LBW		NBW			*p*-Value		
Genes	RN	NN	RN	NN	SEM	BWC	DT	BWC × DT
Claudin-3	0.578	1.000	0.471	0.546	0.17	0.086	0.126	0.279
ZO1	0.836	1.001	0.734	0.997	0.26	0.835	0.405	0.847
ALP	4.505 ^a^	1.000 ^b^	0.564 ^b^	2.384 ^a,b^	1.22	0.283	0.477	0.031
SI	4.154	1.000	1.038	1.564	1.08	0.226	0.214	0.086
EAAC1	1.968	0.998	3.198	3.877	0.99	0.043	0.881	0.402
B0AT1	1.228	1.001	1.867	2.696	0.41	0.007	0.464	0.203
PEPT1	1.545	1.000	2.519	2.593	0.39	0.002	0.533	0.414
SGLT1	1.627 ^a,b^	1.000 ^b^	1.598 ^a,b^	2.127 ^a^	0.27	0.048	0.853	0.038
ASCT2	0.531	1.000	0.154	0.459	0.36	0.189	0.267	0.812

ALP; alkaline phosphatase, SI; sucrase-isomaltase, SGLT1; sodium/glucose cotransporter 1, EAAC1; excitatory amino acid transporter 1, ASCT2; glutamine transporter, B^0^AT1; neutral amino acid transporter, PepT1; peptide transporter 1, ZO-1; zonula occludens-1, CLDN3; claudin 3, CycA; Cyclophilin-A. LBW; low birth weight, NBW; normal birth weight, RN; restricted nutrition; NN; normal nutrition, BWC; birth weight category, DT; dietary treatment. ^a,b^ Lowercase letters within a row represent significant treatment interaction (*p* < 0.05).

**Table 3:** Jejunal nutrient transporter and tight junction mRNA transcript abundance at 56 d post-weaning.

	LBW		NBW			*p*-Value		
Genes	RN	NN	RN	NN	SEM	BWC	DT	BWC × DT
Claudin 3	0.952	1.510	0.250	2.171	0.37	0.955	0.002	0.075
ZO1	0.596	2.098	0.420	3.293	0.52	0.338	<0.001	0.200
ALP	0.688 ^b^	7.321 ^b^	0.656 ^b^	20.934 ^a^	3.11	0.041	<0.001	0.041
SI	2.594 ^b^	4.977 ^b^	1.097 ^b^	15.718 ^a^	2.28	0.052	<0.001	0.012
EAAC1	0.574	1.371	1.593	1.931	0.53	0.148	0.294	0.669
B0AT1	1.264	2.776	0.685	4.455	0.69	0.435	<0.001	0.116
PEPT1	1.114	1.235	1.820	1.150	0.49	0.532	0.581	0.427
SGLT1	0.679	0.552	0.595	0.971	0.20	0.413	0.539	0.222
ASCT2	0.556	1.520	0.130	3.018	0.61	0.388	0.004	0.127

ALP; alkaline phosphatase, SI; sucrase-isomaltase, SGLT1; sodium/glucose cotransporter 1, EAAC1; excitatory amino acid transporter 1, ASCT2; glutamine transporter, B^0^AT1; neutral amino acid transporter, PepT1; peptide transporter 1, ZO-1; zonula occludens-1, CLDN3; claudin 3, CycA; Cyclophilin-A. LBW; Low birth weight, NBW; normal birth weight, RN; restricted nutrition; NN; normal nutrition, BWC; birth weight category, DT; dietary treatment. ^a,b^ Lowercase letters within a row represent significant treatment interaction (*p* < 0.05).

## Data Availability

Data are available from the corresponding author upon reasonable request.
